# Tumor Immune Microenvironment Characteristics and Their Prognostic Value in Non-Small-Cell Lung Cancer

**DOI:** 10.3389/fonc.2021.634059

**Published:** 2021-03-03

**Authors:** Dan Su, Gao Wu, Ran Xiong, Xiangxiang Sun, Meiqing Xu, Yide Mei, Xianning Wu

**Affiliations:** ^1^School of Nursing, Anhui Medical University, Hefei, China; ^2^Department of Thoracic Surgery, The First Affiliated Hospital of USTC, Division of Life Sciences and Medicine, University of Science and Technology of China, Hefei, China; ^3^School of Life Sciences, University of Science and Technology of China, Hefei, China

**Keywords:** tumor immune microenvironment, non-small-cell lung cancer, CD8 T cells, PD1, CD38

## Abstract

**Introduction:**

Cancer progression is determined not only by the malignant behavior of tumors but also by the immune microenvironment. The tumor immune microenvironment also plays a pivotal role in determining the clinical response of non-small-cell lung cancer (NSCLC) to immunotherapies. To understand the possible mechanisms and explore new targets in lung cancer immunotherapy, we characterized the immune profiles in NSCLC patients.

**Methods:**

Seventy-one NSCLC patients who underwent radical resection were selected. The immune cell composition in paired tumor and adjacent normal lung tissues was tested by flow cytometry. The associations of tumor immune microenvironment characteristics with clinicopathological factors and overall survival were analyzed. Kaplan–Meier curves and Cox proportional hazards models were used to determine differences in survival.

**Results:**

Compared with adjacent normal lung tissues, an increased proportion of CD45^+^ hematopoietic-derived cells, CD4^+^ T cell subtypes, Tregs and B cells was observed in tumor samples with a reduced frequency of myeloid cell populations. There was no significant increase in total CD8^+^ T cells, but both PD1^+^ and CD38^+^ CD8^+^ T cells were significantly enriched in tumor samples and statistically significantly associated with tumor size. In addition, positive CD38 expression was highly correlated with PD1 positivity. A high proportion of CD8^+^ T cells and a low percentage of PD1^+^ CD8^+^ T cells were statistically significantly associated with better survival in stage II and III patients, whereas a low frequency of CD38^+^ CD8^+^ T cells was statistically significantly associated with better survival in all patients and identified as an independent prognostic factor (*p*=0.049).

**Conclusion:**

We profiled the immune cells in the tumor tissues of NSCLC patients using flow cytometry. The results revealed significant enrichment of infiltrating immune cells. A strong correlation was identified between CD38 and PD-1 expression on CD8^+^ T cells in tumors. CD8^+^ T cells and their subtypes play a critical role in the prediction of prognosis.

## Introduction

Lung cancer remains the leading cause of cancer-related mortality worldwide (1). With the success of immune checkpoint blockade in the treatment of non-small-cell lung cancer (NSCLC) patients, many combinational immunotherapy strategies are currently being explored in clinical trials (2). However, the response rate remains unsatisfactory (3). In the treatment of lung cancer patients, immunotherapies mainly aim to restore T cell mediated anti-tumor immunity or suppress the pro-tumor activities in the tumor microenvironment (TME).

The TME contains abundant tumor cells and immune cells and is a central regulator of malignant tumor progression (4). Previous studies revealed that the densities of specific tumor-infiltrating lymphocytes (TILs), such as CD8^+^ and CD4^+^ cells, were associated with cancer prognosis (5, 6). CD8^+^ T cells are one of the key biomarkers that predict the efficacy of immune checkpoint therapy (7). Moreover, myeloid cells are heterogeneous immune cells that belong to the innate immune system. Among myeloid cells, macrophages and dendritic cells (DCs) are well known for their ability to regulate T cell responses, and they might play an important role in cancer progression. Additionally, other factors affect anti-tumor immunity through the TME, such as CD38 and the exhausted T cell marker programmed death-1 receptor (PD-1) (8). CD38 overexpression was reported to be a potential mechanism of acquired resistance to PD-1/PD-L1 blockade by suppressing CD8^+^ T cell proliferation and inducing their differentiation into exhaustive CD8^+^ T cells (9). The immune status of the TME is particularly complex, and its prognostic value has not been fully elucidated.

The cell composition in the TME of NSCLC significantly contributes to cancer progression and the sensitivity of tumors to immune therapies, but the underlying mechanisms remain unclear. Understanding the homeostasis of the immune system, particularly the diversity and roles of TILs in the TME, may help to identify the possible mechanisms. The present work aimed to determine whether the immune microenvironment in NSCLC might influence clinical outcomes. We characterized the immune profiles in NSCLC patients by flow cytometry and analyzed the abundance and prognostic value of different immune subsets in tumors.

## Methods

### Patient Selection and Specimens

Seventy-one patients with NSCLC who underwent radical resection(R0) at the First Affiliated Hospital of USTC from April to December 2017 were selected. Paired tumor and adjacent normal lung tissues were collected from patients undergoing lung surgery. All protocols were reviewed and approved by the Ethics Committee of the First Affiliated Hospital of USTC.

### Sample Collection and Preparation

In this study, both fresh surgically resected lung tumor samples and paired adjacent normal tissues (n = 71) were processed into single-cell suspensions using an enzymatic digestion method. Cells were then stained with fluorescently-labeled antibodies before flow cytometry analysis. Briefly, surgical tissue samples were mechanically dissociated with a gentleMACS Octo Dissociator and Human Tumor Dissociation Kit (Miltenyi Biotec) in accordance with the manufacturer’s instructions. Red blood cells were lysed using Red Blood Cell Lysing Buffer (Sigma-Aldrich). Cell suspensions were washed with RPMI1640 medium containing 10% FBS (GIBCO) and filtered through a 70-μm cell strainer (Falcon). Trypan Blue-stained cells were counted with a hemocytometer.

### Antibody Staining for Flow Cytometry

For flow cytometry as described before (10), single cells from tumor tissues and paired adjacent normal tissues were washed and stained with Fixable Viability Stain 780 (BD Biosciences) for cell viability measurements. Then, cells were washed with stain buffer (BD Biosciences) and blocked with human Fc block (BD Biosciences) to block Fc receptors. Cells were then stained with fluorophore-labeled anti-human mAbs. For intracellular staining, cells were treated with the Foxp3/Transcription Factor Staining Buffer Set (eBioscience) and then incubated with mAbs against the corresponding intracellular proteins. Stained samples were washed, fixed with 4% paraformaldehyde (Affymetrix) and subsequently analyzed on a Flow Cytometer (BD Biosciences). Flow cytometry gating strategy was showed in **Figure S1**. Frequency of immune subsets based on the following phenotypical markers, CD4 T cells (CD3^+^CD4^+^), CD8 T cells (CD3^+^CD8^+^), B cells (CD19^+^), NK cells (CD56+), Macrophages/DCs (CD11b^+^CD11c^high^HLA-DR^+^), MDSCs/Granulocytes (CD11b^+^CD11c^low^HLA-DR^-^), M-MDSC (CD11b^+^CD11c^low^HLA-DR^-^CD14^+^CD15^-^), Bregs (CD19^+^CD38^+^CD24^+^), and Tregs (CD3^+^CD4^+^CD25^+^CD127^low^).

### Follow-Up and Data Collection

The clinicopathological and postoperative follow-up data of all patients were collected retrospectively from medical databases. The pathological staging was based on the criteria of the AJCC pTNM classification (eighth edition). The postoperative follow-up procedure included radiological examination on chest every 3 months for the first 2 years and then every 6 months for the next 3 years, and whole body examination emphasizing on head, bone and adrenal gland for each year. Follow-up data collection was performed by trained investigators during inpatient visits, outpatient visits or telephone calls. Overall survival (OS) was defined as the time from the surgery date to death or the last follow-up date.

### Statistical Analyses

Statistical analyses were performed using SPSS version 20.0 (Statistical Package for the Social Sciences, Chicago, IL). Normally distributed data are shown as the mean ± standard deviation. Independent sample t-tests and one-way analysis of variance (ANOVA) were used to compare the means between groups, and Pearson’s correlation was used to analyze the relationship between CD38 and PD-1 expression on CD8^+^ T cell subsets. The Kaplan–Meier method was used to estimate survival. Differences between survival curves were analyzed using the log-rank test. A multivariate analysis of prognostic factors was performed using a Cox proportional hazards model and stepwise procedure. Hazard ratios (HRs) and 95% confidence intervals (CIs) were generated. All statistical tests conducted were two-sided, and *p*<0.05 was considered statistically significant.

## Results

### Immune Profiles in the Tumor Microenvironment of Non-Small-Cell Lung Cancer

**Figure 1:**
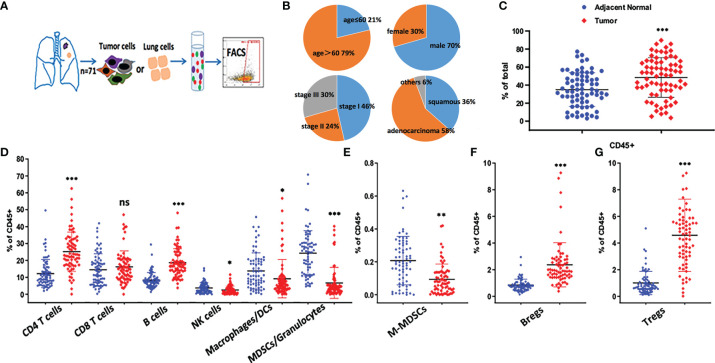
Immune profiling of NSCLC tumor microenvironment. **(A)** Overview of the study design. Fresh surgical resections of adjacent normal lung and tumor tissues were collected from patients. Tissue samples were digested into single cell suspensions for the following staining. The data was analyzed either by flow cytometry. **(B)** Demographic information of the patients was shown. In total, 71 paired samples were collected. **(C)** The percentage of CD45^+^ cells were compared between adjacent normal lung and tumor tissue samples, ***p < 0.001 by paired t-test. Dot plots show Mean ± SD. **(D–G)** Frequency of immune subsets based on the following phenotypical markers, CD4 T cells (CD3^+^CD4^+^), CD8 T cells (CD3^+^CD8^+^), B cells (CD19^+^), NK cells (CD56+), Macrophages/DCs (CD11b^+^CD11c^high^HLA-DR^+^), MDSCs/Granulocytes (CD11b^+^CD11c^low^HLA-DR^-^), M-MDSC (CD11b^+^CD11c^low^HLA-DR^-^CD14^+^CD15^-^), Bregs (CD19^+^CD38^+^CD24^+^), Tregs (CD3^+^CD4^+^CD25^+^CD127^low^), ns *p* > 0.05, *p < 0.05, **p < 0.01, ***p < 0.001 by paired t-test. Dot plots show Mean ± SD.

To characterize the tumor immune microenvironment of NSCLC patients, freshly resected tumors and paired adjacent normal lung tissues were collected (**Figure 1A**). Seventy-one paired samples were analyzed by flow cytometry. Clinical information, including gender, age, pathological type and stage, was collected and summarized in **Figure 1B**. A quality control standard for sample preparation was used, and only processed samples with ≥70% viability were stained. For flow cytometry analysis, at least 5×10^4^ events (LOQ, limitation of quantification) were required to calculate the MFI values of each immune cell population.

We designed three staining panels to distinguish between tumor cells, myeloid cells and T cells. Compared with adjacent normal lung tissues, an increased proportion of CD45^+^ hematopoietic-derived cells was observed in tumor samples, and CD4^+^ T cells were found to be the largest cell population in the TILs (**Figures 1C, D**). In adjacent normal lung samples, a higher frequency (40%) of myeloid cell populations (MDSCs/granulocytes, macrophages/DCs) was identified compared with tumor samples (**Figure 1D**). Our data indicated that at least 18% of CD45^+^ cells were CD19^+^ B cells, which was similar to the amount of CD8^+^ T cells in tumors.

Because M-MDSCs and Bregs are well-known regulatory cells that secrete suppressive cytokines, such as TGF-β and IL-10, the changes in these immune populations were evaluated (**Figures 1E, F**). Bregs were found to be significantly enriched in the TME. In contrast, fewer M-MDSCs were present in tumors. Furthermore, Tregs were found to be highly enriched in tumors (**Figure 1G**). which may be the major suppressive cells to protect tumor cells from being attacked by the immune system. Based on the abundance of these suppressive cells in the lung TME, targeting Tregs or Bregs may potentially restore anti-tumor immunity.

### Association Between the Tumor Immune Microenvironment and Clinicopathological Variables

Tumor specimens from 71 patients were analyzed by flow cytometry. The proportion of total immune cells (CD45^+^) and each subtype was compared according to the clinicopathological characteristics of patients, including gender, age, pathological type, tumor size, stage of involved lymph nodes and pathological stage. Overall, no consistent association between the percentage of TIL subtypes and major clinicopathological variables was found. However, in the subgroup analysis, increased PD1^+^ and CD38^+^ CD8^+^ T cells were statistically significantly associated with tumor size (*p* = 0.004 and *p* = 0.036, respectively) (**Table 1**).

**Table 1:** Association between the tumor immune microenvironment and clinicopathological variables.

Variables	Cases	CD45+ cells (%)	p value	CD4+ T cells (%)	p value	B cells (%)	p value	NK cells (%)	p value	Macrophages/DCs(%)	p value	G-MDSCs (%)	p value	M-MDSCs (%)	p value	Bregs (%)	p value	Tregs (%)	p value	CD8+ T cells(%)	p value	PD-1+ CD8+ T cells(%)	p value	CD 38+ CD8+ T cells(%)	p value	
Gender																										
Male	50	47.38 ± 23.04	0.547	24.65 ± 12.23	0.551	16.72 ± 6.97	0.718	2.48 ± 2.30	0.792	9.04 ± 1.38	0.874	6.61 ± 10.30	0.193	0.090 ± 0.08	0.618	2.58 ± 1.88	0.081	19.75 ± 9.18	0.079	16.99 ± 10.91	0.264	49.30 ± 20.73	0.746	29.39 ± 18.19	0.849	
Female	21	50.88 ± 19.84		26.48 ± 10.45		19.21 ± 9.84		2.64 ± 2.52		9.52 ± 14.77		4.53 ± 4.70		0.102 ± 0.12		1.83 ± 0.71		15.92 ± 5.39		14.2 ± 4.56		47.47 ± 23.63		30.31 ± 18.89		
Age																										
≤60	15	49.56 ± 21.40	0.743	29.25 ± 11.41	0.131	20.12 ± 10.45	0.197	2.79 ± 2.89	0.476	4.68 ± 3.08	0.084	7.04 ± 9.53	0.871	0.098 ± 0.11	0.858	2.17 ± 1.94	0.619	15.68 ± 6.05	0.128	15.67 ± 8.49	0.826	48.89 ± 25.53	0.978	26.87 ± 12.88	0.510	
>60	56	47.76 ± 22.64		24.10 ± 11.62		16.74 ± 7.89		2.46 ± 2.21		10.39 ± 12.45		6.61 ± 9.06		0.093 ± 0.09		2.41 ± 1.59		19.40 ± 8.79		16.29 ± 9.85		48.72 ± 20.51		30.41 ± 19.49		
Pathological type																										
Squamous cell carcinoma	26	49.94 ± 23.43	0.312	23.64 ± 12.76	0.157	16.43 ± 5.94	0.305	2.90 ± 2.75	0.416	11.16 ± 12.86	0.340	9.37 ± 12.23	0.511	0.080 ± 0.08	0.797	2.74 ± 2.00	0.303	20.87 ± 10.93	0.618	18.69 ± 12.09	0.246	52.83 ± 19.45	0.086	29.47 ± 19.00	0.998	
Adenocarcinoma	41	48.44 ± 21.92		27.02 ± 11.10		18.36 ± 6.18		2.05 ± 1.60		6.48 ± 7.63		4.83 ± 6.24		0.091 ± 0.09		2.17 ± 1.45		17.30 ± 6.36		14.99 ± 7.73		44.56 ± 21.78		29.78 ± 18.35		
Other	4	38.23 ± 14.59		16.43 ± 4.28		N/A		N/A		N/A		N/A		N/A		1.74 ± 0.62		N/A		N/A		65.32 ± 22.96		29.71 ± 17.06		
Tumor size																										
≤3cm	37	48.47 ± 22.29	0.984	27.21 ± 12.85	0.130	17.24 ± 10.11	0.897	2.29 ± 1.98	0.365	6.99 ± 10.04	0.091	7.61 ± 9.74	0.383	0.099 ± 0.09	0.630	2.44 ± 1.77	0.667	17.39 ± 7.18	0.200	14.82 ± 7.34	0.218	41.82 ± 20.52	0.004	26.42 ± 18.48	0.036	
>3cm	34	48.36 ± 22.14		22.99 ± 10.00		17.70 ± 12.09		2.80 ± 2.71		11.56 ± 12.35		5.71 ± 8.35		0.088 ± 0.09		2.27 ± 1.55		19.95 ± 9.46		17.63 ± 11.39		56.30 ± 20.13		33.18 ± 17.63		
Status of involved lymph nodes																										
N0	43	48.12 ± 23.75	0.663	24.27 ± 11.50	0.585	16.66 ± 9.87	0.216	2.47 ± 1.96	0.663	7.53 ± 9.72	0.120	6.67 ± 8.64	0.563	0.106 ± 0.10	0.346	2.25 ± 1.39	0.058	18.27 ± 8.36	0.405	16.39 ± 9.66	0.962	47.61 ± 22.09	0.856	28.87 ± 18.34	0.344	
N1	13	48.84 ± 19.90		25.07 ± 13.65		17.10 ± 10.12		3.05 ± 2.85		14.93 ± 16.52		8.74 ± 12.04		0.082 ± 0.07		3.28 ± 2.69		21.34 ± 9.66		16.05 ± 11.09		50.83 ± 18.40		36.06 ± 20.68		
N2	15	52.40 ± 19.27		27.93 ± 10.75		20.09 ± 13.27		2.26 ± 2.98		8.93 ± 9.46		5.01 ± 7.64		0.069 ± 0.08		1.86 ± 0.75		17.25 ± 7.26		15.60 ± 8.24		50.27 ± 23.23		26.38 ± 15.53		
Pathological stage																										
I	33	47.03 ± 23.36	0.644	25.34 ± 11.96	0.942	16.28 ± 8.94	0.089	2.30 ± 1.95	0.733	7.54 ± 10.28	0.519	7.36 ± 9.45	0.487	0.105 ± 0.09	0.503	2.09 ± 1.22	0.054	17.28 ± 8.84	0.092	16.04 ± 8.87	0.387	45.07 ± 23.35	0.397	27.99 ± 19.67	0.755	
II	17	53.08 ± 20.05		24.35 ± 11.08		17.29 ± 8.96		2.83 ± 2.14		11.04 ± 12.60		7.88 ± 10.97		0.095 ± 0.09		3.36 ± 2.65		22.47 ± 8.72		18.65 ± 11.96		52.92 ± 16.99		31.92 ± 11.90		
III	21	46.83 ± 21.00		25.63 ± 12.25		19.47 ± 6.77		2.65 ± 3.08		10.27 ± 12.13		4.71 ± 6.63		0.075 ± 0.08		1.97 ± 0.69		17.59 ± 6.63		14.35 ± 8.31		51.17 ± 21.61		30.46 ± 20.65		

### Characterization of CD8 T Cell Subtypes

**Figure 2:**
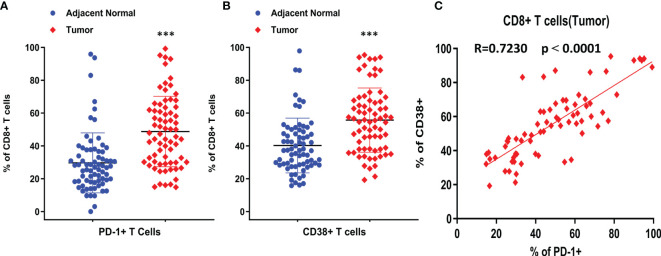
The expression and correlation of PD-1 and CD38 on CD8+ T cells. **(A)** Percentage of PD-1^+^ CD8^+^ T cells was compared between adjacent normal and tumor tissues. **(B)** Percentage of CD38^+^ CD8^+^ T cells was compared between adjacent normal and tumor tissues. **(C)** The correlation between CD38 and PD-1 on CD8^+^ T cells from tumor samples. Pearson r was shown as R, ***p < 0.001 by paired t-test. Dot plots show Mean ± SD.

CD8^+^ T cells are one of the key biomarkers that predict the efficacy of immune checkpoint therapy, and they play an important role in anti-tumor immunity. However, in this study, there was no significant increase in the number of total CD8^+^ T cells in tumors. To investigate the characteristics of CD8^+^ T cell subtypes, we analyzed the expression of T cell exhaustion markers, such as PD-1, and the results showed that 50% of CD8^+^ T cells in tumors were PD1^+^ in almost half of the patients (**Figure 2A**). CD38 is also an immune molecule expressed on the surface of several immune cells, and CD38^+^ CD8^+^ T cells were reported to have strong immunosuppressive capabilities. Our data demonstrated that over 55% of CD8^+^ T cells in the TME were CD38^+^ in at least half of the NSCLC patients (**Figure 2B**). Compared with normal lung samples, both PD-1^+^ and CD38^+^ CD8^+^ T cells were significantly enriched in tumor samples. Furthermore, we found that the positivity of CD38 was highly correlated with PD-1 positivity (**Figure 2C**), suggesting CD38 as an additional marker of exhausted tumor-infiltrating T cells.

### Prognostic Significance of Immune Cells in Tumor Microenvironment

The median follow-up duration was 32.5 months (range, 9.0–42.0 months) for all patients, and the follow-up rate was 94%. The proportion of immune cells was categorized as high and low according to the mean value. Variables of the immune microenvironment and clinicopathological features were used to evaluate the prognosis by Kaplan–Meier survival analysis. Univariate analysis revealed that the tumor size (*p*=0.042), status of involved lymph nodes (*p*<0.001), pathological stage (*p*<0.001) and the proportion of CD38^+^ CD8^+^ T cells (*p*=0.004) were statistically significantly associated with the 3-year survival rate, whereas the other variables were not related to the 3-year overall survival rate (*p* for all > 0.05) (**Table 2**). An increased proportion of macrophages/DCs or Tregs was associated with poor survival, although this result was not statistically significant (*p*=0.082 and *p*=0.076, respectively).

**Table 2:** Univariate survival analyses of patients with NSCLC.

Variables	Cases	Median survival time (months) (95%CI)	3-year cancer-specific survival rate (%)	p value
Gender				
Male	50	35.3 (32.1–38.5)	70.6	0.538
Female	21	33.2 (27.5–38.8)	64.4	
Age				
≤60	15	33.8 (26.9–40.7)	73.3	0.901
>60	56	34.9 (31.8–37.9)	67.2	
Pathological type				
Squamous cell carcinoma	26	33.7 (28.8–38.6)	61.4	0.361
Adenocarcinoma	41	36.1 (32.7–39.4)	74.5	
Other	4			
Tumor size				
≤3cm	37	38.7 (34.2–42.0)	78.2	0.042
>3cm	34	32.5 (28.1–37.0)	60.1	
Status of involved lymph nodes				
N0	43	39.9 (37.6–42.2)	92.1	0.000
N1	13	30.0 (23.4–36.6)	42.3	
N2	15	25.1 (18.2–32.1)	33.3	
Pathological stage				
I	33	41.2 (39.8–42.7)	96.3	0.000
II	17	36.0 (30.3–41.7)	74.0	
III	21	26.1 (20.0–32.2)	38.1	
CD45^+^ cells				
low	33	33.5 (29.3–37.7)	63.9	0.267
high	38	35.5 (31.7–39.3)	70.7	
CD4^+^ T cells				
low	40	35.3 (31.7–38.9)	68.8	0.605
high	31	33.5 (29.0–38.0)	64.5	
B cells				
low	39	35.8 (31.8–38.4)	68.7	0.711
high	32	32.4 (27.7–35.9)	65.5	
NK cells				
low	40	36.2 (32.8–39.5)	70.8	0.344
high	31	30.1 (27.9–37.2)	62.3	
Macrophages/DCs				
low	53	35.6 (32.5–38.8)	73.2	0.082
high	18	30.9 (24.9–37.0)	47.1	
G-MDSCs				
low	55	34.8 (31.6–38.0)	69.1	0.594
high	16	33.6 (27.7–39.5)	59.7	
M-MDSCs				
low	43	34.5 (30.9–38.1)	66.7	0.954
high	28	34.5 (29.9–39.1)	67.4	
Breg				
low	46	35.6 (32.2–39.0)	73.8	0.187
high	25	32.6 (27.7–37.5)	54.8	
Treg				
low	45	35.9 (32.5–39.4)	76.4	0.076
high	26	32.0 (27.3–36.8)	50.4	
CD8^+^ T cells				
low	43	33.5 (29.6–37.3)	63.5	0.397
high	28	36.1 (32.0–40.0)	72.1	
PD-1^+^ CD8^+^ T cells				
low	37	36.5 (32.3–39.9)	76.4	0.114
high	34	32.4 (28.0–36.8)	56.4	
CD 38^+^ CD8^+^ T cells				
low	43	37.6 (34.5–40.7)	81.7	0.004
high	28	30.1 (25.4–34.9)	45.4	
				

**Figure 3:**
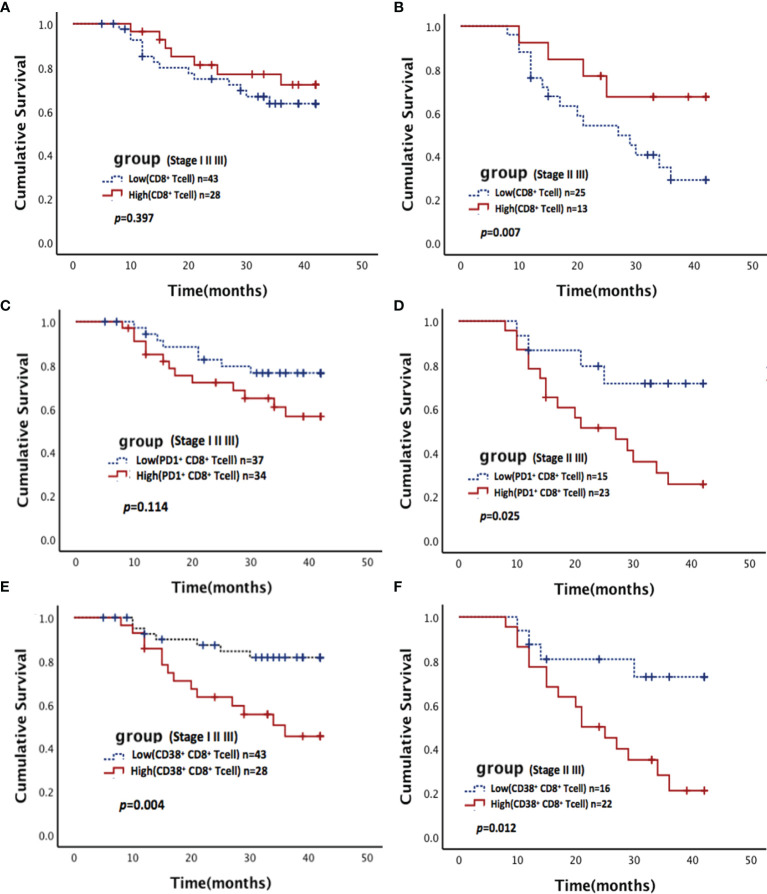
Association between CD8+ T cells and their subsets with survival in NSCLC. Kaplan-Meier estimates of overall survival (OS) stratified by pathological stage according to CD8^+^ T cells **(A, B)**, PD-1^+^ CD8^+^ T cells **(C, D)**, and CD38^+^ CD8^+^ T cells **(E, F)**. **(A, C, E)** for all stage patients (including stages I, II, and III); **(B, D, F)** for middle and advanced stage patients (including stages II and III).

As mentioned above, 46% of patients were stage I, and there was only 1 death during the follow-up period. The survival rates stratified by pathological stage were assessed. A high proportion of CD8^+^ T cells and a low frequency PD1^+^ CD8^+^ T cells were statistically significantly associated with better survival in stage II and III patients, but no significant differences in survival were found among all patients (**Figures 3A–D**). A low proportion of CD38^+^ CD8^+^ T cells was statistically significantly associated with better survival in all patients and stage II and III subgroups (**Figures 3E, F**).

In the multivariate analysis, a Cox proportional hazards model was used to identify the independent predictors of OS, which were adjusted for all immune microenvironment and clinicopathological factors. Details of the results were presented in **Table 3**. The analyses revealed that the status of involved lymph nodes (*p*<0.001), pathological stage (*p*<0.001) and proportion of CD38^+^ CD8^+^T cells (*p*=0.049) were independent prognostic factors and significantly correlated with OS.

**Table 3:** Multivariate survival analyses of patients with NSCLC.

Variables	RR (95%CI)	*p* value
Status of involved lymph nodes	2.662 (1.080–6.560)	<0.001
Pathological stage	4.198 (2.179–8.088)	<0.001
CD 38^+^ CD8^+^ T cells	2.449 (1.004–5.971)	0.049

## Discussion

Cancer progression is determined not only by the malignant behavior of tumors but also by the immune microenvironment. The tumor immune microenvironment also plays a pivotal role in determining the clinical response of NSCLC patients to immunotherapies. To understand the possible mechanisms and explore new targets for lung cancer immunotherapy, we characterized the immune profiles in NSCLC patients by flow cytometry and determined the abundance of different immune cells in the TME. Compared with adjacent normal lung tissues, more CD45^+^ hematopoietic-derived cells were observed in tumor samples, and CD4^+^ T cells were the largest cell population in TILs, suggesting that TILs play an important role in tumor progression. These data are consistent with previous findings (11) and confirm that the microenvironment of NSCLC tumors is enriched with immune cells. However, there were no differences among clinicopathological subgroups, and no prognostic value was identified. This finding is likely attributed to the complexity of the TME. Different immune cell populations have various biological roles in tumor regulation (12).

Although there were no differences in CD8^+^ T cells between tumor and normal tissues, we found that CD8^+^ T cells were associated with better prognosis in advanced stage NSCLC patients, which was consistent with previous studies (5, 13). CD8^+^ T lymphocytes are a well-established group of effector T cells with potent cytotoxic effects in cancer. And exhausted CD8^+^ T cells are a distinct cell lineage that have decreased effector function and proliferative capacity, partly caused by overexpression of inhibitory receptors such as PD-1 (14). In lung cancer animal models, the anti-PD-L1 treatment group showed an increase in exhausted CD8^+^ T cells (14). High variability in the abundance of PD-1^+^ CD8^+^ T cells was observed, and these cells were increased in tumors larger than 3 cm. An increased proportion of PD-1^+^ CD8^+^ T cells was correlated with poor survival in stages II and III patients, which strongly suggested the effect of CD8^+^ T cell exhaustion on anti-tumor immunity. Although the relationship between PD-1 expression and prognosis remains controversial, which may be associated with its essential modulation for effector T cells and their direct role in overcoming the immunosuppressive TME (15).

Extensive evidence suggests that tumor-infiltrating CD8^+^ T cells positively contribute to anti-tumor immunity; however, the role of tumor-infiltrating B cells remains controversial. Studies have reported a positive prognostic effect, generally similar to that of CD8^+^ T cells (16, 17). We also identified that B cells were enriched in the TME at a level about 4.5-fold higher than previously reported (18). This large difference in B cell proportions may be due to differences in tumor tissue sample collection because B cells are mainly present at the tumor periphery. The function of these B cells is still unknown, which has resulted in different opinions on whether immunotherapies should be designed to enhance or inhibit these cells. Bregs enriched in tumors are defined by their production of cytokines, such as IL-10, IL-35, and TGF-β, and are known to suppress inflammation and T-cell immunity (19). A lack of phenotypical markers for Bregs and the plasticity of B cells in response to the tumor milieu has limited the research progress on targeting B cells in cancer immunotherapy (20).

In NSCLC tumors, fewer macrophages and DCs were present in the TME compared with adjacent normal lung tissues which was consistent with previous research (10), indicating chronic inflammation or disrupted tumor homeostasis in these NSCLC patients. MDSCs are divided into polymorphonuclear (PMN) and monocytic (M) MDSCs (21). Although specific phenotypical markers for MDSCs in lung tumors are debatable, the most important criterion defining MDSCs is their ability to suppress T cells, and M-MDSCs have a more extensive suppressive ability compared with PMN-MDSCs (22). However, due to the particularly rare presence of MDSCs in the tumors of NSCLC patients, the depletion of MDSCs using an antibody may not be sufficient to reverse the immune-suppression status of the TME and restore an anti-tumor one.

CD38 is a type II transmembrane glycoprotein and multifunctional ectoenzyme involved in calcium mobilization, cell adhesion and signaling pathways (23).In this study, more than 55% of the tumor-infiltrating CD8^+^ T cells in NSCLC patients were CD38^+^, which was significantly higher than the proportion in normal lung tissues. It was surprising that increased CD38^+^ CD8^+^ T cells but not Tregs in the TME were associated with poor prognosis in NSCLC as Tregs may be the main suppressive cells to protect tumor cells from being attacked by the immune system (24). Multivariate analysis confirmed the critical role of CD38^+^ CD8^+^ T cells in the prediction of prognosis, which may help to evaluate prognosis more accurately when combining this information with the pathological stages. However, the potential functions of CD38 on other immune cells have not yet been fully elucidated and require further study.

A recent study indicates that the overexpression of CD38 on T cells after PD-1/PD-L1 blockade may contribute to acquired resistance by suppressing CD8^+^ T cell proliferation and inducing their differentiation into exhaustive CD8^+^ T cells, and the combination of CD38 and PD-L1 blockade substantially reduces primary tumor burden and metastasis compared with PD-L1 blockade alone *in vivo*. The inhibitory mechanism of CD38 on CD8^+^ T cells was found to be mediated by the adenosine receptor signaling pathway (9). Another study revealed that PD-1 blockade in unprimed or suboptimally primed CD8 cells induces resistance through the induction of PD-1^+^ CD38^+^ CD8^+^ cells that is reversed by optimal priming. PD-1^+^ CD38^+^ CD8^+^ cells serve as a predictive and therapeutic biomarker for anti-PD-1 treatment (25). These findings, together with the strong correlation between CD38 positivity and PD1 positivity on CD8^+^ T cells found in our study, suggests that CD38 is likely an important player in the regulation of T cell suppression in lung cancer patients.

This study investigated frequency of immune cells based on a large number of specimens comparing malignant and adjacent normal tissues. As we discussed, the composition and characteristics of the tumor immune microenvironment are important in determining the anti-tumor immune response and may affect tumor therapy for NSCLC. However, when patients received radical resection, there is no tumor immune microenvironment. Anti-tumor immune response in this situation has not been fully elucidated. And the association between immune characteristics of tumor and adjuvant therapy are not clear.

This study has several limitations. First, the sample size was small. Except for squamous carcinoma and adenocarcinoma, only 4 patients had other pathological types, including 3 cases adenosquamous carcinoma and 1 cases large cell lung cancer. Immune profile data and scatter plot showed no obvious abnormal deviation but without statistical verification. In further study we should collected more cases of rare pathological type to draw the whole immune map of NSCLC. Second, there were too many early-stage patients, and the longest follow-up was 42 months, which may result in the overlooking of valuable prognostic factors, such as Tregs. Third, correlation between molecular diagnosis and immune microenvironment characteristics have not been evaluated, because of incomplete molecular information such as EGFR mutation, ALK fusion. Forth, although our preliminary findings suggested a potential prognostic role of CD8^+^ T cells and their subtypes and more studies focused on PD-1, the potential functions of CD38 on other immune cells has not been fully elaborated which should be identified in further studies.

## Conclusion

We profiled the immune cells in the tumor tissues of NSCLC patients using flow cytometry. The results revealed significant enrichment of infiltrating immune cells. A strong correlation was identified between CD38 and PD-1 expression on CD8^+^ T cells in tumors. CD8^+^ T cells and their subtypes play a critical role in the prediction of prognosis.

## Data Availability Statement

The original contributions presented in the study are included in the article/**Supplementary Material**. Further inquiries can be directed to the corresponding author.

## Ethics Statement

The studies involving human participants were reviewed and approved by the Ethics Committee of the First Affiliated Hospital of University of Science and Technology of China. Written informed consent for participation was not required for this study in accordance with the national legislation and the institutional requirements.

## Author Contributions

All authors contributed to the article and approved the submitted version. DS, MX, and XW contributed to the study design. YM and XW were responsible for the interpretation of the results. GW, RX, and XS contributed to the statistical analysis. DS and XW wrote the manuscript.

## Funding

This work was supported by Anhui Provincial Natural Science Foundation (1808085QH270; 2008085QH428) and the Fundamental Research Funds for the Central Universities (WK9110000121).

## Conflict of Interest

The authors declare that the research was conducted in the absence of any commercial or financial relationships that could be construed as a potential conflict of interest.

## Supplementary Material

Supplementary Figure 1 Gating strategy of Tumor infiltrating cells by flow cytometry. The representative graph to show how to gate different immune subsets based on the phenotypical markers. And isotype and FMO controls samples were also used to validate the baseline of negative populations.
