# Supplementary figures and images for: Tumor Immune Microenvironment Characteristics and Their Prognostic Value in Non-Small-Cell Lung Cancer
Source: Front Oncol. 2021 Mar 3;11:634059. doi: 10.3389/fonc.2021.634059 (PMC7966704; doi:10.3389/fonc.2021.634059)

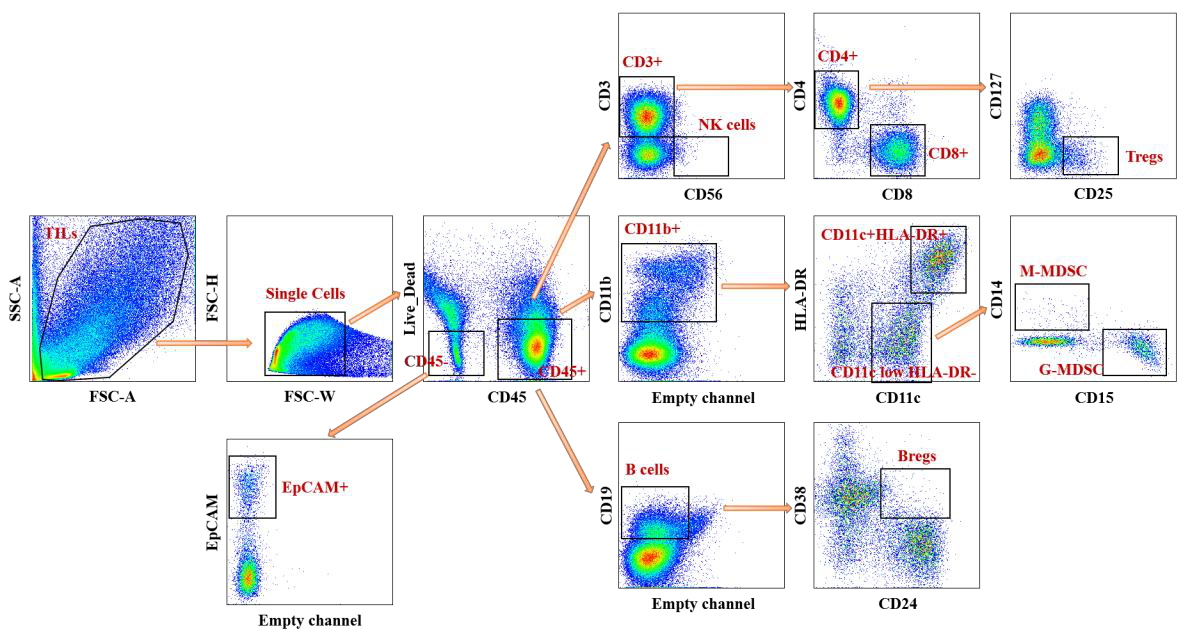

Supplement: Supplementary Figure 1 — Gating strategy of Tumor infiltrating cells by flow cytometry. The representative graph to show how to gate different immune subsets based on the phenotypical markers. And isotype and FMO controls samples were also used to validate the baseline of negative populations. [file Image_1.png]
